# Excretion of Host DNA in Feces Is Associated with Risk of *Clostridium difficile* Infection

**DOI:** 10.1155/2015/246203

**Published:** 2015-05-18

**Authors:** Caroline Vincent, Sudeep Mehrotra, Vivian G. Loo, Ken Dewar, Amee R. Manges

**Affiliations:** ^1^Department of Microbiology and Immunology, McGill University, Montreal, QC, Canada H3A 2B4; ^2^McGill University and Génome Québec Innovation Centre, Montreal, QC, Canada H3A 0G1; ^3^Department of Human Genetics, McGill University, Montreal, QC, Canada H3A 1B1; ^4^The Research Institute of the McGill University Health Centre, Montreal, QC, Canada H3H 2R9; ^5^School of Population and Public Health, The University of British Columbia, Vancouver, BC, Canada V6T 1Z3

## Abstract

*Clostridium difficile* infection (CDI) is intricately linked to the health of the gastrointestinal tract and its indigenous microbiota. In this study, we assessed whether fecal excretion of host DNA is associated with CDI development. Assuming that shedding of epithelial cell increases in the inflamed intestine, we used human DNA excretion as a marker of intestinal insult. Whole-genome shotgun sequencing was employed to quantify host DNA excretion and evaluate bacterial content in fecal samples collected from patients with incipient CDI, hospitalized controls, and healthy subjects. Human DNA excretion was significantly increased in patients admitted to the hospital for a gastrointestinal ailment, as well as prior to an episode of CDI. In multivariable analyses, human read abundance was independently associated with CDI development. Host DNA proportions were negatively correlated with intestinal microbiota diversity. *Enterococcus* and *Escherichia* were enriched in patients excreting high quantities of human DNA, while *Ruminococcus* and *Odoribacter* were depleted. These findings suggest that intestinal inflammation can occur prior to CDI development and may influence patient susceptibility to CDI. The quantification of human DNA in feces could serve as a simple and noninvasive approach to assess bowel inflammation and identify patients at risk of CDI.

## 1. Introduction

In healthy individuals, the intestinal microbiota is characterized by a highly complex and dynamic microbial community which contains as many as 1,000 bacterial species [1]. This microbial community constitutes an important metabolic organ that provides numerous beneficial functions to the host, including the digestion of complex carbohydrates, production of vitamins, maturation of the immune system, regulation of gastrointestinal transit, and stimulation of epithelial cell turnover [2]. The indigenous microbiota also has the ability to outcompete opportunistic microorganisms and enteric pathogens like* Clostridium difficile* through a process known as colonization resistance [3].

Human fecal matter is composed of a mixture of water, undigested food, microorganisms, and epithelial cells released from the walls of the gastrointestinal tract [4]. The desquamation of intestinal epithelial cells can be quantified by measuring the abundance of human DNA excreted in feces. Although the intestinal epithelium undergoes rapid turnover and is completely renewed every 4-5 days [5], typically very low amounts of human DNA can be detected in fecal matter [6]. However, when intestinal homeostasis is perturbed due to the presence of infectious agents or inflammation, greater amounts of damaged and dead epithelial cells are exfoliated from the intestinal wall, resulting in higher quantities of human cells shed in feces [7]. In sequenced-based studies of the fecal microbiome, the excretion of host DNA has not been well-characterized. Studies using directed PCR and sequencing of phylogenetically informative regions of the bacterial 16S ribosomal RNA gene (rDNA) do not interrogate human DNA, and studies based on whole-genome shotgun (WGS) sequencing often apply bioinformatic filters to remove low quality reads, host DNA, and other contaminants prior to analyses.

*C. difficile* is the major etiological agent of infectious diarrhea and pseudomembranous colitis in hospitalized patients. The main risk factor for* C. difficile* infection (CDI) is antibiotic exposure and the overall risk increases with prolonged and combined use of antibiotics [8]. Broad-spectrum antibiotics have profound detrimental effects on the structure and diversity of the intestinal microbiota [9, 10]. These alterations can result in loss of colonization resistance, thereby providing an opportunity for* C. difficile* proliferation.* C. difficile* can also elicit intestinal inflammation during colonization as a way to further disrupt the indigenous microbiota and overcome colonization resistance [11].

Despite advances in infection control practices and the development of new treatment options, there has been a steady increase in the incidence and severity of CDI in the last two decades and outbreaks continue to occur in hospitals and healthcare institutions worldwide [12, 13]. As there is presently no vaccine for CDI, the development of new strategies for early identification of high-risk patients and earlier diagnosis of patients undergoing CDI would aid in infection prevention and patient management. In order to achieve this task, there is a need for an improved understanding of the intestinal ecosystem, including factors that maintain intestinal homeostasis and colonization resistance in the face of constantly changing environmental pressures.

The objective of this study was to investigate the relationship between intestinal epithelial cell shedding, microbiota composition, and subsequent development of nosocomial CDI. We used WGS sequencing to compare the proportions of human DNA and evaluate bacterial content in fecal samples obtained from (i) patients prior to the onset of CDI (cases), (ii) hospitalized controls, and (iii) nonhospitalized healthy subjects. Our results provide evidence that the excretion of high quantities of host DNA in feces is a general outcome of intestinal inflammation and is associated with CDI risk in hospitalized patients.

## 2. Materials and Methods

### 2.1. Subject Recruitment and Sample Collection

Between September 2006 and May 2007, a total of 599 hospitalized patients were enrolled in a prospective cohort study at the Royal Victoria Hospital in Montreal. A detailed description of the study population and definitions are available in Loo et al. [14]. A single rectal swab was obtained from each patient within 7 days of admission to the hospital. A questionnaire was administered to all study subjects to collect information on demographics, known risk factors for CDI, and use of various medications in the 8 weeks prior to hospital admission and during hospitalization. During the study period, 31 patients experienced one or more CDI episodes. After excluding patients with a history of previous CDI, fecal samples collected prior to CDI diagnosis were available for 18 patients (cases). Thirty-six controls were selected from patients who did not develop CDI, either during hospitalization or up to 60 days after discharge. Case patients were matched to controls in a 1 : 2 ratio based on sex, age (±5 years), and date of hospitalization (±2 months). All participants provided informed written consent. The human subjects' protocols for the cohort and case-control studies were approved by the Royal Victoria Hospital Internal Review Board as well as the McGill University Institutional Review Board (BMB 05-014). As nonhospitalized healthy controls, we included intestinal microbiome sequence data from 88 adults generated as part of the Human Microbiome Project (HMP) [6]. To ensure our sequencing data was comparable to that of the HMP, we also included two fecal samples obtained from nonhospitalized healthy adults living in Montreal.

### 2.2. WGS Sequencing and Data Analysis

Fecal DNA was extracted using the DNA IQ System (Promega) and subjected to whole-genome amplification using the illustra GenomiPhi V2 DNA Amplification Kit (GE Healthcare) according to the manufacturers' protocols. Due to limited amounts of fecal material available on the rectal swabs, whole-genome amplification was necessary in order to ensure sufficient DNA quantities for subsequent steps. Multiplexed DNA libraries were prepared according to a previously described protocol [15]. WGS sequencing was performed at the McGill University and Génome Québec Innovation Centre and the Illumina HiSeq 2000 instrument was used to generate 150-nucleotide sequence reads. Eleven to 15 samples were pooled in each sequencing lane and we obtained a median of 18.0 million reads per sample (range, 6.4–91.4 million). In each fecal sample, the proportion of reads derived from the human genome was determined with BMTagger [16]. In order to assess microbial diversity, we retrieved all reads containing the V1–V3 reverse primer sequence (which targets a segment of the 16S rRNA gene) [17] and their 3′ sequences in order to obtain 55-mers originating with the primer sequence. We compiled the occurrence and frequency of these 16S rDNA motifs to measure the diversity of each sample using the inverse Simpson index. MetaPhlAn [18] was used to infer genus-level taxonomic abundances and assess the presence of* C. difficile* in fecal samples. All samples contained at least 1,000 reads with a hit to MetaPhlAn's marker database.

### 2.3. Statistical Analyses

Kernel density estimation and all other statistical analyses were performed with the R software [19]. LEfSe [20] was used to perform linear discriminant analysis and identify bacterial genera that discriminate between three categories of human DNA abundance.

## 3. Results and Discussion

**Figure 1:**
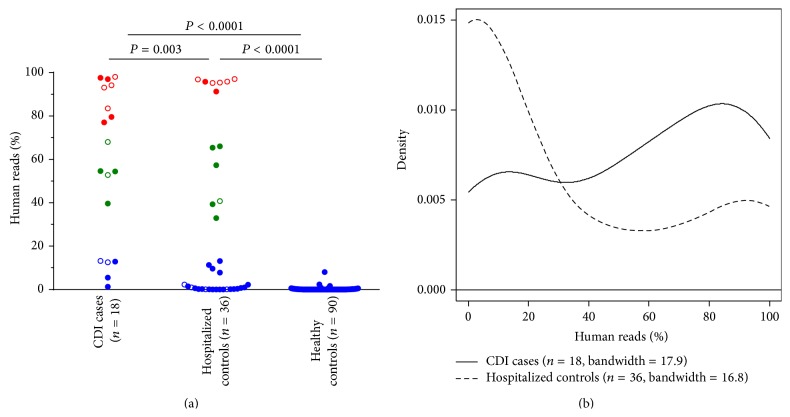
Distribution of human read proportions in fecal samples from CDI cases, hospitalized controls, and healthy subjects. (a) Scatter plot showing the proportions of human reads in each subject group. Patients admitted to the hospital for gastrointestinal problems (8 cases and 10 controls) are displayed as open circles. Three subgroups representing low (<30%, shown in blue), intermediate (30–70%, shown in green), and high (>70%, shown in red) amounts of human DNA were apparent from the sample distributions. *P* values were determined by Mann-Whitney *U* test. (b) Kernel density plot showing the distribution of human read proportions in CDI case and hospitalized control groups.

We compared the proportions of human DNA in fecal samples collected from 18 CDI cases, 36 hospitalized controls, and 90 nonhospitalized healthy controls. Among case patients, the median interval of time between stool collection and CDI diagnosis was 10.5 days. The proportion of human reads detected in fecal samples differed significantly between the three subject groups (Figure 1(a)). In hospitalized patients (cases and controls), the proportion of human reads was highly variable, with values ranging from 0 to 98%. In contrast, the vast majority (95.5%) of stool samples from healthy subjects, including two samples sequenced in this study, contained less than 1% of human reads. Overall, samples from incipient CDI cases contained significantly higher proportions of human reads than samples from hospitalized and healthy controls (*P* ≤ 0.003 by Mann-Whitney *U* test; Figure 1(a)). We acknowledge that variations in sample collection procedures (i.e., whole stool for healthy subjects versus rectal swabs for hospitalized patients) may have contributed to the observed differences in human read proportions between subject groups. To better evaluate and compare the complex underlying distributions of the incipient CDI case and hospitalized control groups, we generated a kernel density plot (Figure 1(b)). While fecal samples with low amounts (<30%) of human DNA were predominant in hospitalized controls (64% of samples), samples from incipient CDI cases displayed a shift towards intermediate (30–70%) and high (>70%) levels of host DNA content (72% of samples had >30% of human reads).

A review of patient records indicated that 18 (8 cases and 10 controls) out of 54 patients were admitted to the hospital for a gastrointestinal surgery or infection (other than CDI) (Figure 1(a), shown as open circles). The fecal samples obtained from patients admitted for a gastrointestinal ailment had significantly higher proportions of human reads compared to patients admitted for other reasons (*P* = 0.01 by Mann-Whitney *U* test). Increased excretion of human DNA has also been observed in patients with active ulcerative colitis and colorectal cancer, as well as in patients undergoing pelvic radiotherapy [21–25]. Taken together, these observations indicate that increased fecal excretion of host DNA is a general marker of intestinal insult.

In order to distinguish between the contributions of non-CDI related gastrointestinal ailment (based on hospital admission information) and incipient CDI and how they affect the level of human DNA excretion, we performed a multivariable analysis and found that human read abundance remains significantly associated with subsequent CDI development when admission for gastrointestinal problems is controlled for in the logistic regression model (*P* ≤ 0.02). This indicates that while excretion of large amounts of human DNA is a general indicator of gastrointestinal insult and inflammation, it is also independently associated with CDI development. We were particularly intrigued by the increase in the proportion of CDI patients with intermediate levels (30–70%) of human reads in their fecal sample and consider two scenarios that could explain their importance in CDI. In the first case, the observation of intermediate levels of human DNA may reflect a low-grade, chronic inflammatory state. If so, then the presence of intermediate to high levels of human DNA provides another measure of intestinal health that aids in assessing patient risk to CDI. The presence of inflammation in the gut can have deleterious effects on the intestinal microbiota and may create a permissive environment for* C. difficile* colonization, thereby increasing patient susceptibility to CDI. Alternatively, intermediate levels of host DNA excretion may reflect an early inflammation stage elicited by* C. difficile* colonization and thus represent an early sign of CDI. Although we cannot distinguish whether increased shedding of intestinal epithelial cells is a risk marker for CDI or whether it is inherent to CDI development, the observation that only 2 out of 18 cases had DNA sequences corresponding to* C. difficile* in their stool and the absence of a relationship between human read abundance and time to CDI diagnosis (*r* = 0.30, *P* = 0.22 by Spearman correlation) supports the idea that increased amounts of human DNA in feces is a risk marker for CDI. Larger studies using longitudinal sample collection are needed to clarify this question.

**Figure 2:**
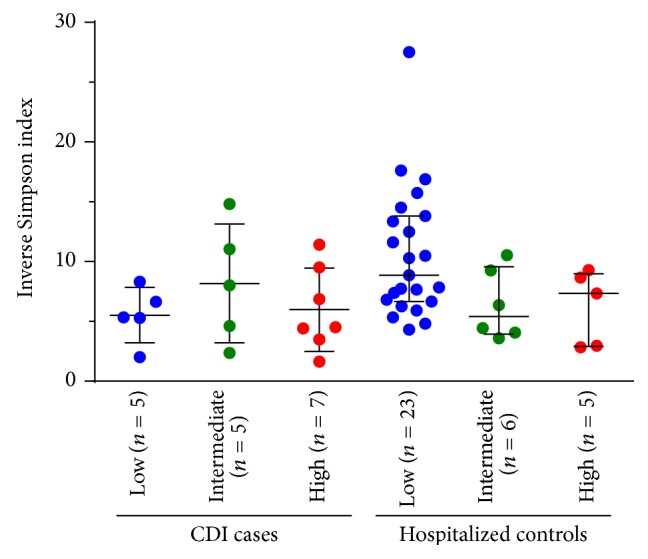
Intestinal microbiota diversity in hospitalized cases and controls as a function of host DNA abundance. Each patient group was divided into three subgroups with low (<30%, shown in blue), intermediate (30–70%, shown in green), and high (>70%, shown in red) proportions of human reads. One sample from the CDI group and two samples from hospitalized controls were excluded because they contained less than 15 reads with a 16S rDNA motif. Horizontal lines represent the median and interquartile range.

Since intestinal epithelium integrity, intestinal inflammation, and resident microbiota are intricately related, we assessed the diversity and composition of the fecal microbiota with respect to human DNA content. As there was little to no human DNA in samples from healthy subjects, we restricted our microbiome analyses to the hospitalized cases and controls. In order to estimate bacterial diversity from WGS datasets, we employed an rDNA data mining approach. 16S rDNA motifs (defined as 55-mer DNA segments starting with the V1–V3 primer sequence) were recovered after partitioning human DNA sequences. Three samples (one from a CDI case and two from hospitalized controls) with very high human DNA content (≥95.8% of reads) contained less than 15 rDNA motifs and were excluded from the diversity analyses. Of the 51 remaining samples, four contained more than 95.8% of human reads but still had enough rDNA motifs to allow a reliable estimation of bacterial diversity. In hospitalized controls, we observed a trend of decreasing microbial diversity with increasing proportions of human DNA (*r* = −0.50, *P* = 0.003 by Spearman's rank correlation test; Figure 2). This trend was not observed in CDI cases (*r* = −0.14, *P* = 0.6). Patients with incipient CDI exhibited overall low levels of microbial diversity, a feature that is already associated with CDI susceptibility [26]. Nonetheless, intestinal inflammation could be the common link between increased epithelial cell shedding and reduced microbial diversity.

Bacterial genomes typically range in size from 0.5 to more than 9 Mb and contain between 1 and 15 copies of the 16S rRNA gene [27, 28], so the naive expectation for pure bacterial samples would be to observe approximately one 16S rDNA read per Mb of sequence data. Within our population of 54 hospitalized patients and two healthy subjects, the frequency of 16S rDNA motifs ranged from 0.1 to 0.7 occurrences per Mb of total sequence data after accounting for host DNA. In a comparable analysis of a variety of individually sequenced bacterial species (*Listeria monocytogenes*,* Vibrio fluvialis*,* Salmonella enterica*, and* Campylobacter jejuni*) with genome sizes ranging from 1.8 to 6.7 Mb and 16S rDNA copy numbers ranging from 1 to 8, rDNA motifs were observed at a frequency of 1 to 3 occurrences per Mb. Therefore, 16S rDNA motif recovery rates differed by an order of magnitude between purified bacterial cultures and fecal microbiome samples. Whereas fecal DNA samples have been considered to be predominantly of bacterial origin, our results imply that other sources of DNA that are not of bacterial or human origin (e.g., food, fungi, protozoa, bacteriophages, and other viruses) account for a substantial fraction of the fecal microbiome and likely play a role in the interplay between the microbial community and the host. Nielsen et al. have also recognized that the microbiome is composed of a wide variety of microbes and approaches assessing the full genetic diversity are needed [29]. Even with the paucity of robust tools to identify these other sources of DNA, the use of methods that survey the whole community rather than focusing only on bacteria, such as WGS sequencing, offers a starting point for taking a census of the entire microbiome.

**Figure 3:**
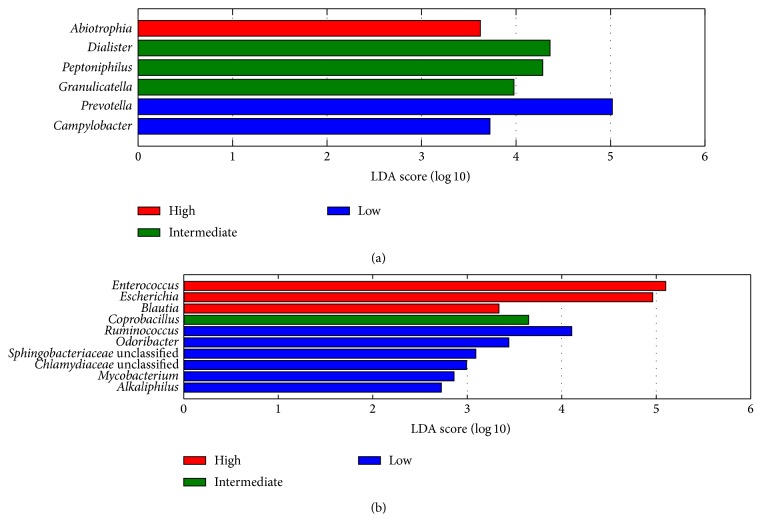
Bacterial genera that discriminate between low (<30%, shown in blue), intermediate (30–70%, shown in green), and high (>70%, shown in red) proportions of human DNA in (a) CDI cases and (b) hospitalized controls. The histograms show bacterial genera with an absolute log linear discriminant analysis (LDA) score of at least 2.0.

We also examined whether specific bacterial genera are associated with fecal excretion of human DNA in hospitalized patients. In the incipient CDI group,* Prevotella* was more abundant in patients with low levels of host DNA. This genus is a common member of the fecal microbiota in adults [30]. In the hospitalized control group,* Enterococcus* and* Escherichia* were enriched in patients with high proportions of host DNA, while* Ruminococcus* and* Odoribacter* were depleted (Figure 3(b)). Increased levels of* Enterococcus* and* Escherichia* have been previously associated with reduced colonization resistance, intestinal inflammation, and Crohn's disease [31–34]. Reduced abundance of* Ruminococcus* and* Odoribacter* has also been observed in patients with inflammatory bowel disease [31, 34]. These genera are important producers of short-chain fatty acids, which are metabolized by epithelial cells in the colon and play a major role in the regulation of colonocyte differentiation and proliferation [35, 36]. Therefore, a decrease in their abundance may have detrimental effects on the integrity of the intestinal epithelium and on the regulation of inflammation.

Although we did not directly measure the presence of intestinal inflammation in our population, a growing body of evidence (including this study) suggests that host DNA excretion is a general outcome of intestinal insult [21–24]. While earlier studies have directly targeted exfoliated colonocytes or human DNA via quantitative PCR, we believe WGS sequencing provides a comprehensive and unbiased way to assess fecal DNA content. Moreover, as the measurements are obtained from fecal samples, it provides a noninvasive and straightforward approach for monitoring intestinal health status. Whereas our study generated millions of sequences to support the characterization of intestinal microbiota, the monitoring of host DNA content could be achieved with much smaller datasets, thereby reducing costs and decreasing the time to results.

Our results also have implications for how different genomic approaches for studying the intestinal microbiome can affect informed consent and human subject protocols. Fecal samples used in WGS studies, especially for individuals with gastrointestinal diseases, provide information on the host genome as well as the microbiome. In instances of elevated host cell excretion, where host DNA constitutes more than 50% of the sequencing data, consent protocols should indicate that the data produced may be comparable to directly sequencing the individual's genome.

## 4. Conclusions

We have shown that fecal excretion of abundant quantities of human DNA is significantly associated with incipient CDI and appears to be an outcome of intestinal inflammation. High levels of human DNA were associated with a reduction of intestinal microbiota diversity, an increase in opportunistic microorganisms, and a depletion of short-chain fatty acid-producing bacteria. Fecal excretion of host DNA should be investigated as a potential clinical marker of intestinal inflammation and CDI risk.
